# Relationship between gut microbiota and Chinook salmon (*Oncorhynchus tshawytscha*) health and growth performance in freshwater recirculating aquaculture systems

**DOI:** 10.3389/fmicb.2023.1065823

**Published:** 2023-02-07

**Authors:** Ruixiang Zhao, Jane E. Symonds, Seumas P. Walker, Konstanze Steiner, Chris G. Carter, John P. Bowman, Barbara F. Nowak

**Affiliations:** ^1^Institute for Marine and Antarctic Studies, University of Tasmania, Newnham, TAS, Australia; ^2^Cawthron Institute, Nelson, New Zealand; ^3^Institute for Marine and Antarctic Studies, University of Tasmania, Hobart, TAS, Australia; ^4^Centre for Food Safety and Innovation, Tasmanian Institute of Agriculture, Hobart, TAS, Australia

**Keywords:** Chinook salmon, gut microbiota, swim bladder, recirculating aquaculture system, growth performance

## Abstract

Gut microbiota play important roles in fish health and growth performance and the microbiome in fish has been shown to be a biomarker for stress. In this study, we surveyed the change of Chinook salmon (*Oncorhynchus tshawytscha*) gut and water microbiota in freshwater recirculating aquaculture systems (RAS) for 7 months and evaluated how gut microbial communities were influenced by fish health and growth performance. The gut microbial diversity significantly increased in parallel with the growth of the fish. The dominant gut microbiota shifted from a predominance of *Firmicutes* to *Proteobacteria,* while *Proteobacteria* constantly dominated the water microbiota. *Photobacterium* sp. was persistently the major gut microbial community member during the whole experiment and was identified as the core gut microbiota for freshwater farmed Chinook salmon. No significant variation in gut microbial diversity and composition was observed among fish with different growth performance. At the end of the trial, 36 out of 78 fish had fluid in their swim bladders. These fish had gut microbiomes containing elevated proportions of *Enterococcus, Stenotrophomonas*, *Aeromonas,* and *Raoultella*. Our study supports the growing body of knowledge about the beneficial microbiota associated with modern salmon aquaculture systems and provides additional information on possible links between dysbiosis and gut microbiota for Chinook salmon.

## 1. Introduction

The gut microbial communities have been studied in over 145 species of fish (Sullam et al., 2012; Llewellyn et al., 2016; Perry et al., 2020; Nikouli et al., 2021; Luna et al., 2022) suggesting their important role in fish, including synthesizing digestive enzymes, producing vitamins, and enhancing the maturation of the intestine-related immune system (Yukgehnaish et al., 2020). In marine fish, gut microbial communities are primarily dominated by four bacterial phyla, *Proteobacteria*, *Firmicutes*, *Fusobacteria*, and *Actinobacteria*, while at the species and strain level, there is significant diversity, resulting from fish species differences and inter-individual variability (Egerton et al., 2018). Aquaculture now supplies over 45% of fish-based food products worldwide (Longo et al., 2019) and the interest in the gut microbiota of salmonids is accelerating due to their significant economic importance in aquaculture (Minich et al., 2020; Nguyen et al., 2020; Zhao et al., 2020, 2021; Steiner et al., 2021, 2022). The gastrointestinal (GI) tract is also an important site for infections (Xiong et al., 2019) and in that respect the GI microbiome is a potential biomarker for stress (Perry et al., 2020). Therefore, a better understanding of the host-microbiota interactions is essential to maintain fish health in the long term (Gauthier et al., 2019).

An imbalance or disorder in the types and numbers of gut microbiota taxa present in the GI tract may lead to gut dysbiosis, which can impact host health. Gut dysbiosis can be categorized into three types: a loss of beneficial microorganisms, an expansion of pathobionts or potentially problematic microorganisms, or a reduction in microbial diversity (Petersen and Round, 2014). A loss of beneficial microorganisms and a reduction in microbial diversity can inhibit nutrient absorption and impact growth performance, thus reducing aquaculture productivity (Vargas-Albores et al., 2021). For example, *Pseudomonas plecoglossicida* infection causes an irreversible dysbiosis in the gut microbiota of large yellow croaker (*Larimichthys crocea*), resulting in a disease-like gut bacterial community and increasing mortality (Li C. et al., 2020). The overgrowth of gut opportunistic bacteria (*Vibrio*, *Aeromonas*, and *Shewanella*) and the depression of beneficial bacteria (*Cetobacterium*) in diseased Crucian Carp (*Carassius auratus*) are found to be associated with the occurrence of the “red-operculum” disease (Li et al., 2017). Additionally, haemorrhagic septicaemia in farmed Chinese sturgeon (*Acipenser sinensis*) (Di et al., 2018) and yersiniosis in farmed rainbow trout *(Oncorhynchus mykiss)* (Mesías et al., 2019) are all suggested to be correlated with dysbiosis. However, it is still unclear if changes in the gut microbiota are a cause or result of these diseases. Gut microbiota composition data has been used to identify biomarker taxa potentially enabling diagnosis of changes in the health status of Atlantic salmon (Bozzi et al., 2021). They found the dominance of an unclassified *Mycoplasma* genus in the gut of healthy farmed Atlantic salmon might be correlated with defending against pathogens and promoting growth performance and suggested to use this distinct mollicute taxon as a biomarker to monitor the health status of farmed salmonids in real-time *via* non-invasive sampling procedures (Bozzi et al., 2021). The presence of *Mycoplasma* sp. has also been reported in healthy farmed Chinook salmon (Zhao et al., 2020). Whether aquaculture farm managers can obtain useful information by monitoring the relative abundance of the *Mycoplasma* sp. is actually feasible let alone feasible requires much further investigation. Fundamentally there is the possibility other bacterial taxa can supplant this taxon and have a similar role (Klakegg et al., 2020).

Microbial relationships have been studied in various models that operate at different scales, such as linear correlation models between different taxa through co-occurrence networks of the major microbial community members (Poudel et al., 2016; Ganz et al., 2017). Based on co-occurrence networks, alignment-based (Faisal et al., 2014) and alignment-free (Wang J. et al., 2018) methods have been developed to demonstrate the relationship alteration between different conditions, such as health and disease. However, these strategies are unable to quantify the alterations of relationships between key microbes, especially *in vivo*. Targeting the exact species that contribute to the community variation, along with the quantification of certain microbial relationships, could enhance the prediction of dysbiosis and the diagnosis of diseases (Liu Z. et al., 2021). Such information could help fish farmers trying to pinpoint issues associated with poor fish performance in farms especially where there is limited evidence of infections. Furthermore, investigations into diets where the objective is to maximize beneficial bacterial populations may also have clearer and impactful outcomes.

The development of the 16S rRNA gene-based approach with next-generation sequencing technology has contributed to culture-independent assessments of the composition and diversity of gut microbiota (Ghanbari et al., 2015). Although the attention to the gut microbiota of aquatic animals has increased, the understanding of the gut microbiota of farmed Chinook salmon has lagged behind that of farmed Atlantic salmon. Ciric et al. (2019) surveyed the mid-gut microbiome of farmed Chinook salmon in New Zealand and showed that about 80% of them experiencing thermal stress in summer had gut microbiota dominated by *Vibrio* or other *Vibrionaceae* taxa. Zhao et al. (2020) found that microbial richness and diversity were higher in freshwater farmed Chinook salmon than in those farmed in marine farms, and fish age showed significant effects on the composition of gut microbiota in both freshwater and saltwater habitats compared to water temperature and farming location. For salmon raised in saltwater recirculation aquaculture systems (RAS), the gut microbial communities partially overlapped with the ambient environment, including water and feed (Steiner et al., 2021). More recently, the distinct domination of *Photobacterium* spp. was observed in the hindgut of freshwater farmed Chinook salmon with high fecal scores (Zhao et al., 2021). However, this study mainly assessed microbial composition and diversity. Investigating the alteration in microbial relationships can be used to evaluate how bacterial characteristics change in response to specific health outcomes. Currently, the association between gut microbiota and Chinook salmon health as well as growth performance remains poorly defined.

We evaluated the association between gut microbiota and fish health as well as the growth performance of farmed Chinook salmon, raised in freshwater recirculating aquaculture systems (RAS). To track the gut microbiota changes, we surveyed the microbial communities in Chinook salmon digesta in parallel to measures of fish growth and health for 7 months after transfer from the hatchery. We investigated the salmon gut microbial composition and diversity to understand the potential for gut dysbiosis and its association with fish growth performance. Further, we investigated the relationship alterations in abundant microbiota between fish at different sampling time points, growth performance phenotypes and health statuses. The main aim was to provide a more comprehensive picture of the gut microbiota of freshwater Chinook salmon under relatively stable culture conditions and discover critical microbiota associated with fish health and growth performance.

## 2. Materials and methods

### 2.1. Experimental design and fish management

**Figure 1:**
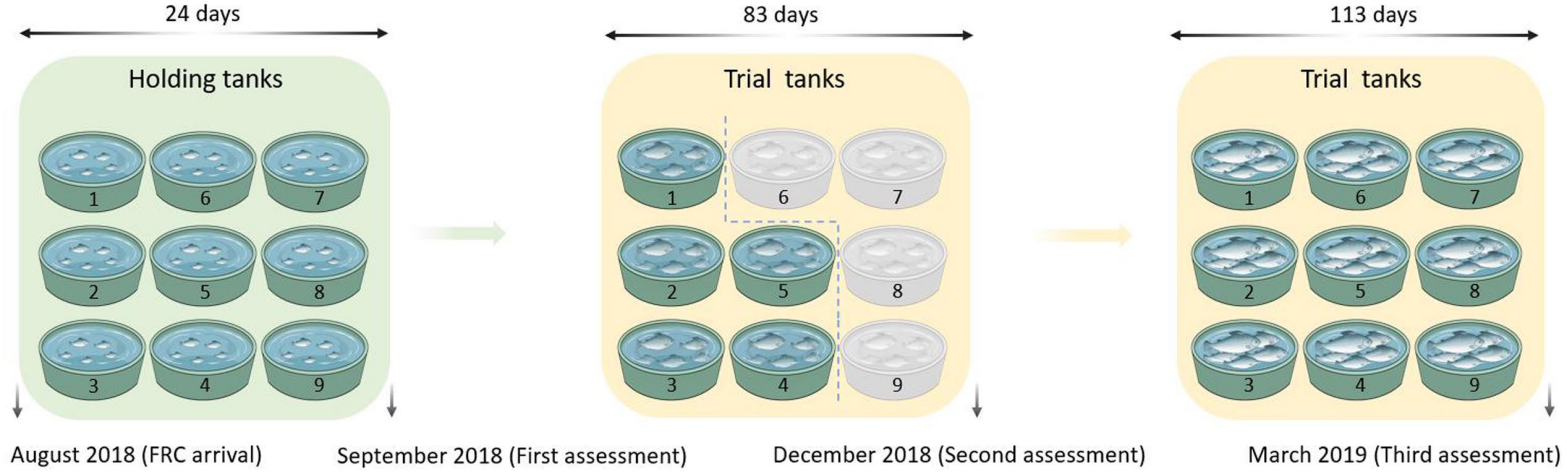
The schematic of the experiment design and timeline. FRC: Finfish Research Centre. Fish from the holding tanks and 17°C trial tanks (green) were included in this study and fish from the 13°C trial tanks (gray) were not used in this study. Six fish per tank were collected and assessed in September 2018 and December 2018. Ten fish per tank were collected and assessed in March 2019. Salmon with empty guts determined by X-radiography were removed and only visually healthy salmon were kept for further assessments based on growth, condition and absence of deformity or external damage (54 fish from September 2018, 30 fish from December 2018, and 78 fish from March 2019). Three water samples of 500 ml each from the same region of each tank were collected along with the digesta samples at three sampling-time points (September 2018, December 2018, and March 2019).

A total of 3,159 all-female juvenile Chinook salmon provided by Mount Cook Alpine Salmon (MCAS) were reared in freshwater RAS at the Finfish Research Centre (FRC) at the Cawthron Aquaculture Park (CAP), Glenduan, New Zealand. The schematic design and detailed information on the experimental system can be found in our previous study (Zhao et al., 2021). Two RAS set-ups comprising 9 tanks designed by Fresh by Design (Moss Vale, New South Wales, Australia) were involved in the present project and each RAS was supplied with fresh water from the local town water supply that had been carbon filtered to remove any residual chlorine. All experimental fish were from a May 2018 stock hatched at the Clearwater Hatchery (Mount Cook Alpine Salmon, New Zealand). In August 2018, they were transferred to the FRC. The experimental fish in the FRC were kept in holding tanks at 15°C for a period of 21 to 24 days before the trial started and were then evenly distributed into trial tanks (8,000 l) recirculated with UV-treated freshwater at 17°C. The maximum density was kept below 25 kg/m^3^ throughout the experimental period. The schematic of the trial design and timeline are shown in Figure 1. The fish were hand-fed daily with 4 mm then 6 mm freshwater diets manufactured specifically for the trial by Ridley Corporation Ltd. (Melbourne, Australia) (crude protein 42.2%, total fat 23.0%, ash 10.0%, moisture 6.2%, carbohydrate 18.6% and energy 22.28 kJ/g).

### 2.2. Fish handling and health assessments

Prior to transfer to the FRC, each fish was tagged with a unique passive integrated transponder (PIT) tag (HID Global, EM4305 684,230, 12 mm glass tags) at Clearwater Hatchery (MCAS). The PIT tag number was scanned into the computer using a microchip tag reader (Avid-Power TracKer VI, Avid Identification Systems, Inc. CA, United States) and used in all data collections to identify the fish and to link data collections together. During the experiment, six fish per tank were collected and assessed at two sampling time points: September 2018 and December 2018. Ten fish per tank were collected and assessed in March 2019. All sampled fish were X-rayed using an Atomscope HFX90V EX9025V portable X-ray Unit (DLC Australia Pty Ltd., Melbourne, Australia) and images were obtained using a Canon CXDI 410C Wireless Cesium Amorphous Silicon digital radiographic receptor (DLC Australia Pty Ltd., Melbourne, Australia). After removing the salmon with empty guts (X-rayed imaging), only visually healthy salmon were kept for further analysis based on growth, condition and absence of deformity or external damage (final sample size: 54 fish from September 2018, 30 fish from December 2018, and 78 fish from March 2019). Before each assessment, fish were anesthetized with 15 ppm AQUI-S for handling (AQUI-S® Aquatic Anesthetic, New Zealand). All fish performance profiles (weight, fork length, and condition factor) were recorded at the time of handling (Table 1) and health profiles (histology, swim bladder status, blood plasma biochemistry and hematology) of 37 fish were recorded at the last assessment (March 2019) (Table 2).

**Table 1:** Growth performance profiles of experimental fish at three sampling timepoints (assessments).

	September 2018	December 2018	March 2019
Number of fish (n)	54	30	78
Weight (g)	165.48 ± 32.21	461.70 ± 62.05	780.01 ± 146.98*
Fork length (mm)	222.80 ± 11.67	296.53 ± 11.48	349.71 ± 19.27*
SGR (w/day%)	1.52 ± 0.19	1.33 ± 0.23	0.54 ± 0.11*
Fulton’s *CF* (K)	1.48 ± 0.11	1.76 ± 0.11	1.81 ± 0.15*

SGR, specific growth rate; CF, condition factor.

Values are represented means ± SEM (standard error of the mean). All results of three sampling time-point groups (September 2018, December 2018 and March 2019) are statistically significant (*p* < 0.05), indicated with an asterisk.

**Table 2:** Growth performance, plasma biochemistry, and hematology profiles of fish with different swim bladder statuses, sampled in March 2019.

	Abnormal swim bladders	Normal swim bladders
Number of fish (n)	17	20
Fish weight (g)	773.94 ± 125.41	775.50 ± 174.22
Fork length (mm)	350.35 ± 18.35	351.90 ± 22.07
Fulton’s *CF* (K)	1.79 ± 0.12	1.75 ± 0.17
SGR (w/day%)	0.41 ± 0.1	0.42 ± 0.14
FS	2.18 ± 1.29	1.60 ± 0.58
Mucous density (n/mm^2^)	134.65 ± 33.68	170.78 ± 57.76
Total protein (g/L)	36.06 ± 5.37	38.70 ± 3.10
Albumin (g/L)	**15.88 ± 2.35**	**17.30 ± 1.55**
Globulin (g/L)	20.24 ± 3.44	21.50 ± 1.91
Alkaline Phosphatase (IU/L)	**15.94 ± 5.26**	**22.55 ± 4.16**
Aspartate aminotransferase (IU/L)	**222.71 ± 69.69**	**325.55 ± 110.98**
Chloride (mmol/L)	140.35 ± 9.37	139.40 ± 4.94
Cholesterol (mmol/L)	**6.28 ± 1.44**	**9.08 ± 1.31**
Creatine phosphokinase (IU/L)	2527.53 ± 3439.96	2513.95 ± 3930.16
Glutamate dehydrogenase (IU/L)	**73.15 ± 26.3**	**133.15 ± 40.73**
Potassium (mmol/L)	4.65 ± 0.55	4.93 ± 0.77
Urea (mmol/L)	**0.84 ± 0.25**	**1.20 ± 0.58**
Phosphate (mmol/L)	3.29 ± 0.38	3.43 ± 0.53
Triglycerides (mmol/L)	1.25 ± 0.94	1.32 ± 0.68
Glucose (mmol/L)	5.10 ± 0.86	5.66 ± 0.97
Cortisol (nmol/L)	178.78 ± 173.92	213.25 ± 116.59
Hemoglobin (g/L)	100.00 ± 9.36	97.05 ± 11.86
White blood cell count (10^9^/L)	19.92 ± 8.38	19.69 ± 6.17
Lymphocytes Absolute (10^9^/L)	19.38 ± 8.33	19.26 ± 6.18
Hematocrit (%)	36.76 ± 2.26	38.23 ± 3.01

Abnormal swim bladders, fish with fluid in their swim bladders; Normal swim bladders, fish with no fluid in their swim bladders; SGR, specific growth rate; FS, fecal score. Values are represented means ± SEM (standard error of the mean). Statistically significant results (*p* < 0.05) are shown in bold.

Fish health was determined based on blood biochemistry and hematology variables, GI tract mucous cell density, and fluid accumulation in their swim bladders. To evaluate the correlation with blood biochemistry and hematology, a total of 19 blood variables (Supplementary Table 1) were measured based on the method outlined by Casanovas et al. (2021). Blood samples were collected (without anti-coagulant) from the caudal vein immediately following euthanasia and plasma samples were obtained from fresh peripheral blood samples after centrifugation (12,045 r/min for 8 min). Plasma samples (500 μl) were snap frozen in liquid nitrogen and sent frozen on dry ice to an International Accreditation New Zealand (IANZ^1^) accredited commercial laboratory (Gribbles Veterinary, Christchurch, New Zealand) for a targeted and quantitative analysis of all biochemistry and hematology analyzes as per International Federation of Clinical Chemistry and Laboratory Medicine (IFCC^2^) recommendations.

To evaluate the association with mucous cell density, hindgut samples were processed using standard protocols for histology and embedded in paraffin (Minich et al., 2020). Sections of 4 μm were cut and one section was used for each fish. The sections were stained with Alcian Blue/ Periodic Acid – Schiff (AB/PAS) at pH 2.5 to quantify mucous cells in the section under a bright field light microscope (Leica DM1000, Hamburg, Germany). Intestinal mucous cells of Chinook salmon collected from March 2019 were counted using established methods (Pittman et al., 2011), and the results were normalized per surface area. After removing poor-quality histology samples, 39 out of 78 fish at the final assessment were divided into 2 clusters (high mucous density fish (HMDF) with a mean density of 220.44 ± 34.70 /mm^2^ and low mucous density fish (LMDF) with a mean density of 126.75 ± 25.65 /mm^2^) by using k-mean cluster analysis (MacQueen, 1967; Supplementary Table 2).

To evaluate the correlation with digestive status, the fecal score (FS) system designed by Zarkasi et al. (2016) for farmed Tasmanian Atlantic salmon was applied to monitor feces (Zhao et al., 2021). This ordered categorical score system (1 to 5, with 5 representing an absence of fecal matter) was used for digestive status comparison based on visual properties. To investigate the association between the gut microbiota and fecal scores, we categorized the fish from March 2019 into four groups (FS1, FS2, FS3, and FS4) based on their FS values.

A swim bladder assessment was conducted at the last sampling event. Fish were carefully dissected and viscera removed to reveal the intact inflated swim bladder. The presence or absence of fluid in the swim bladder was then recorded for each fish.

### 2.3. Fish growth performance phenotype identification

To evaluate the association between growth performance and the gut microbiota, we sampled 78 fish at the final assessment and clustered them based on condition factor (K) and specific growth rate (SGR) by using k-mean cluster analysis (MacQueen, 1967). The K of individual fish was calculated based on the method referred to by Fulton (1911) using the following equation:


$$K=\frac{W\times 100}{L^{3}}$$

- *W* = wet weight (g)
- *L* = fork length (cm)

The SGR (w/day%) of individual fish was calculated based on the method referred to by Koskela et al. (1997) using the following equation:


$$SGR=\frac{\left(\left(\mathrm{lnWf}-\mathrm{lnWi}\right)\times 100\right)}{t}$$

- lnWf = the natural logarithm of the final weight
- lnWi = the natural logarithm of the initial weight
- *t* = time (days) between lnWf and lnWi

### 2.4. Sample collection for microbiota analysis

Digesta samples (less than 0.6 ml) were collected directly from the hindgut of Chinook salmon at three sampling points (September 2018, December 2018, and March 2019). Fish were individually captured from tanks *via* scoop net, euthanised *via* anesthetic overdose with 80 ppm AQUI-S for 7 min (AQUI-S® Aquatic Anesthetic, New Zealand). Digesta samples were placed into cryogenic screw cap tubes and immediately snapped frozen with liquid nitrogen. Tubes were transferred on dry ice and stored in the −80°C freezer at the Cawthron laboratory (Nelson, New Zealand). Between each fish sampling, the surgical tools were cleaned and sterilized with bleach and then 70% (v/v) ethanol to minimize contamination. Three water samples of 500 ml each from the same region of each tank were collected along with the digesta samples. Samples were subsequently filtered using 0.22 μM membrane filters (mixtures of cellulose acetate and cellulose nitrate) (MerckMillipore, United States). Filters were frozen and stored at −80°C until the bacterial DNA was extracted.

### 2.5. DNA extraction and 16S rRNA sequencing

Bacterial DNA was extracted directly from digesta and filter samples using the NucleoSpin Soil kit (Macherey-Nagel, Germany) based on the manufacturer’s instructions. A NanoPhotometer® NP80 spectrophotometer (Implen, Munich, Germany) was used to measure the DNA concentration. Extracted DNA was stored at −80°C until future analysis.

To evaluate the bacterial DNA, 2 × 300 bp pair-ended amplicon sequencing of the V1-V3 region of the 16S rRNA gene was performed using the MiSeq Illumina platform by the Ramaciotti Centre for Genomics (RCG, Kensington, NSW, Australia). The bacterial universal primers 27F (5′ AGA GTT TGA TCM TGG CTC AG 3′) and 519R (5’ GWA TTA CCG CGG CKG CTG 3′) included a 12-base Golay barcode as described by Caporaso et al. (2012) were used for conducting the Polymerase Chain Reaction (PCR). Each step of the molecular analyzes (DNA extraction, PCR, and sequencing library preparation) was performed with sequential workflows to ensure no cross-contamination. The PCR thermocycling conditions were 95°C for 10 min, followed by 40 cycles of 95°C for 30 s, 48°C for 30 s, 72°C for 1 min, and a final extension of 72°C for 7 min. The sequencing data were then trimmed by removing the primer, bar code, and adapter regions using internally developed algorithms by RCG. Pair-ends sequences were joined with the default settings using FASTQ-join (version 1.1.2) (Aronesty, 2011) following trimming, sequences merging, and chimera filtering. Sequences were sorted by individuals and filtered by removing the low-quality reads.

### 2.6. Water and gut microbiota community profiling

Taxonomic analyzes of sequence reads were further processed in the Seed 2 pipeline (Seed v.2.1) (Větrovský et al., 2018). Sequence alignment, denoising, chimera check, and clustering were carried out by using a set of Seed 2 external programs (USEARCH/ v 7.0.1090, MAFFT v 7.215, MOTHUR v1.34.4). The sequences for each cluster were then sorted by length and clustered with a 3% divergence cut-off to define operational taxonomic units (OTU) from centroids. Clusters with fewer than two reads and reads with lengths less than 100 bp were excluded, followed by further clustering at a 3% divergence level using USEARCH to optimize the final consensus sequences accurately and define OTUs. OTUs were classified against the Silva non-redundant 16S rRNA database (SILVA SSU 138, 16 December 2019).

### 2.7. Microbial diversity and composition analyzes

OTU reads were centered log-ratio transformed, and a resemblance matrix was created by calculating the Euclidean distance (Gloor and Reid, 2016). Only relatively high abundance OTUs of which reads make up at least 0.01% in the total dataset were included in diversity analyzes. The microbial diversity was analyzed using PRIMER version 7 with PERMANOVA+ (Primer-E, Ivybridge, United Kingdom). Microbial communities were categorized based on different factors such as sample type, sampling time points, intestinal mucous cell density, swim bladder status, growth performance phenotype, health status and fecal score. Alpha diversity was determined from tabulated sequence data including observed OTU counts and the Shannon diversity index. The analysis of beta diversity was visualized with Principal Coordinates Analysis (PCoA) (Gower, 1966), Non-metric Multi-Dimensional Scaling (nMDS) (Kruskal, 1964), and Canonical Analysis of Principal Coordinates (CAP) plots (Anderson and Willis, 2003). CAP and PERMANOVA analyzes were performed using default settings with 999 permutations. The correlations between microbial diversity and FCR, K, and mucous cell density were evaluated using Pearson correlation coefficient (PCC) analysis (Benesty et al., 2009). The significance of alpha diversity was calculated using a Welch’s t-test and a one-way ANOVA test (Yuen, 1974; Howell, 2012). The multiple pair-wise comparisons of beta diversity were further analyzed using permutation multivariate analysis (PERMANOVA) (Anderson, 2014) in PRIMER v7. A differential abundance analysis was conducted to measure the relative abundance variations in abundant OTUs (> 0.01% total reads) (Gloor et al., 2017). The Welch’s t-test and the Kruskal–Wallis H test were used for measuring the significance of parametric (i.e., fish weight and fork length) and non-parametric data (i.e., microbial richness, diversity, and fecal score) respectively. The significance values were considered significantly different when *p <* 0.05.

### 2.8. Profile monitoring for microbial relationship alteration analysis

The PM2RA framework designed by Liu Z. et al. (2021) for the human microbiome was applied for evaluating the dysbiosis of the Chinook salmon gut microbiome.^3^ PM2RA analysis projects the abundance data of two or more taxa under two conditions into the same space *via* Hoteling’s *T*^2^ statistics and compares the difference in the distribution of T^2^ statistics to represent the relationship alternation (RA) between two conditions. A scoring scheme called profile monitoring (PM) score is specifically designed to quantify RA involving two or more taxa (sub-community) under different conditions (Liu Z. et al., 2021). The more the sub-community alters, the larger the PM score is. In our study, digesta samples that showed significant changes in microbial diversity and composition were subsampled for PM2RA analysis. Furthermore, only abundant OTUs comprising sequences making up >0.01% of the total sequences and detected in higher than 10% of datasets were filtered for the PM2RA analysis. For output visualization, a RA network in which edges denote the corresponding PM score was built by using an open-source software platform Cytoscape.^4^

## 3. Results

### 3.1. Fish health and growth performance

Overall, all experimental salmon sampled for this study were presumed to be healthy based on their external appearance. However, a subgroup (*n* = 36 out of 78 fish) was found fluid in their swim bladder at the final sampling time point (March 2019). Detailed growth performance, plasma biochemistry and hematology profiles are shown in Table 2 for individuals with and without fluid in their swim bladders. After removing poor-quality measurements with missing values, 37 fish with blood biochemistry and hematology profiles were assigned to two clusters by using *k*-mean cluster analysis (MacQueen, 1967). One group of 27 fish with a mean of 87% blood variables within the normal ranges established by Casanovas et al. (2021) for freshwater salmon were clustered into group A, while another group of 10 fish with a mean of 76% blood variables in the normal ranges were clustered into group B (Supplementary Table 1). For relative growth performance, the fish were assigned to two growth performance phenotypes, including 36 high-performing fish (HPF) with relatively higher SGR and K, and 42 low-performing fish (LPF) with relatively lower SGR and K (Supplementary Table 2). Furthermore, based on intestinal mucous cell density, 11 fish were identified as having high mucosal densities (with a mean density of 220.44 ± 34.70 /mm^2^) while 28 fish were identified as having low densities (with a mean density of 126.75 ± 25.65 /mm^2^) (Supplementary Table 2). Fish were categorized into two groups based on plasma biochemistry and hematological data, with group A having most blood variables close to reference levels (*n* = 27) as defined by Casanovas et al. (2021), while 10 fish were categorized as having a higher proportion of the measures outside reference value ranges (Supplementary Table 1). Most fish produced feces with low fecal scores [FS1 (*n* = 27) and FS2 (*n* = 40)] while seven fish had a FS of 3 and only two fish produced pseudofeces (FS 4). Both pseudofeces-producing fish had fluid in their swim bladders.

### 3.2. Microbiome characterization

For the gut microbiota data analysis, a total of 6,215,963 effective reads (Supplementary Table 3) were obtained from digesta samples after filtering singletons and reads assigned to chloroplasts, mitochondria, unassigned bacteria, eukaryotes, and archaea. With the same quality filtration process, we obtained 564,978 effective reads (Supplementary Table 4) for the water microbiota. This contributed to an average of 38,370 reads for digesta samples and 8,188 reads for water samples. Overall, 1876 OTUs from the digesta and 2,325 OTUs from the water were clustered using a similarity threshold of 97%. The raw sequencing data can be found in Supplementary Table 5.

### 3.3. Chinook salmon gut microbiota at different time points

**Figure 2:**
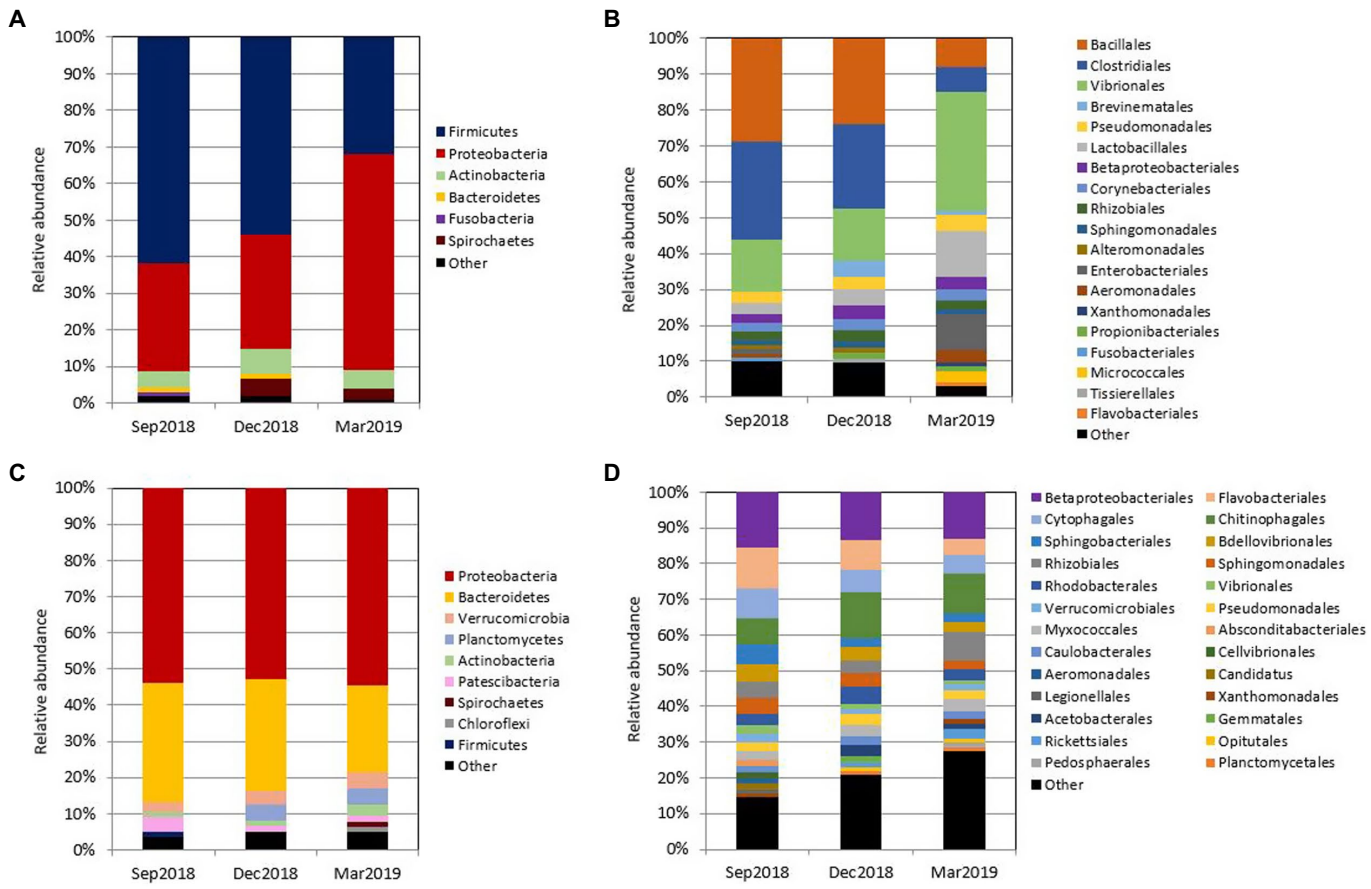
Relative abundance (%) of bacterial phyla and orders in the Chinook salmon gut and rearing water during the different sampling time points. **(A)** Gut microbial composition at the phylum level. **(B)** Gut microbial composition at the order level. **(C)** Water microbial composition at the phylum level. **(D)** Water microbial composition at the order level. Only phyla and orders that are present at relative abundance >1% in at least one sample are shown.

**Figure 3:**
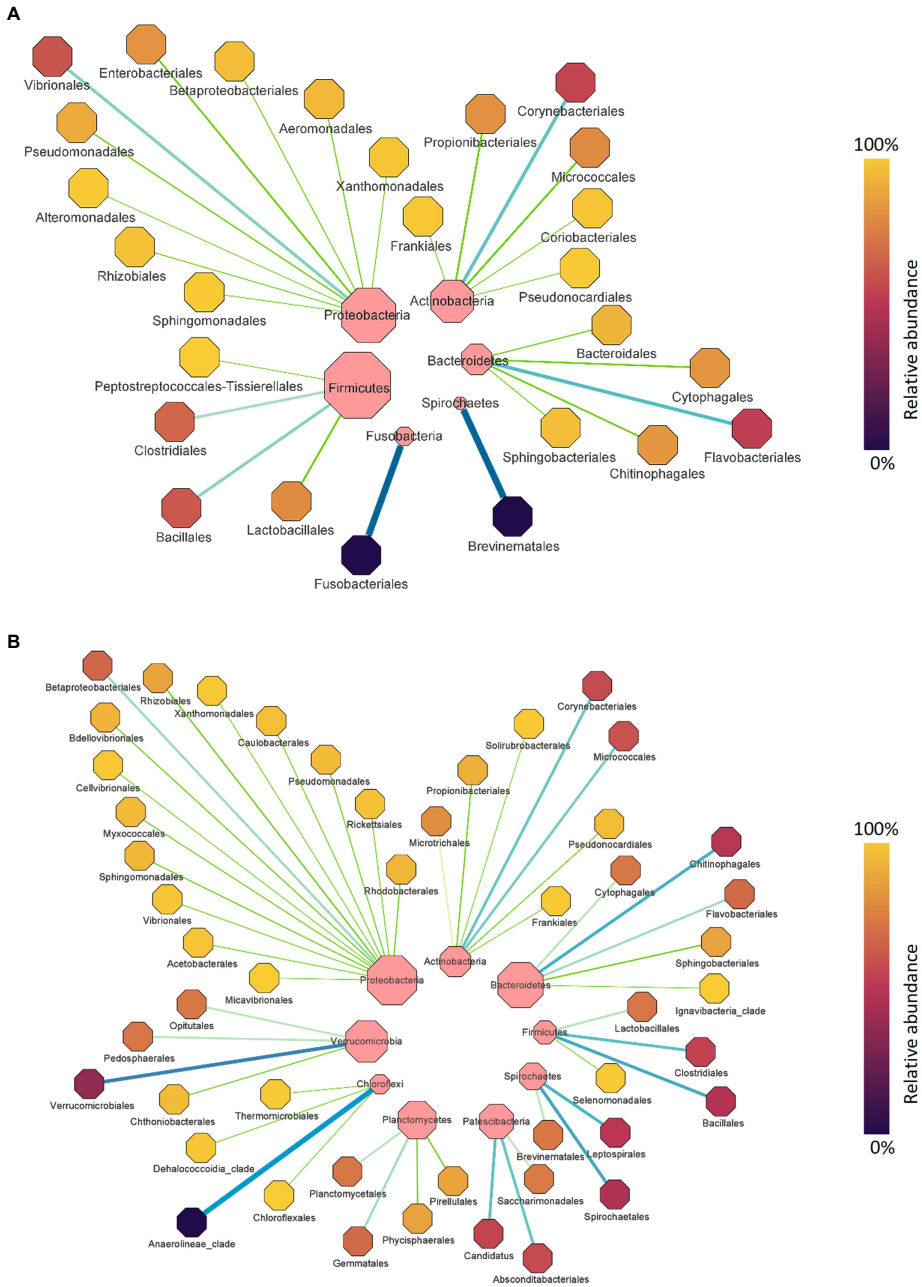
The taxonomic domination of bacterial orders in the Chinook salmon gut **(A)** and rearing water **(B)** during the whole experiment. Only phyla and orders that are present at relative abundance >1% in at least one sample are shown. The size of the central dot represents the relative abundance of each phylum and order, of which larger dots indicate a higher relative abundance and vice versa.

**Figure 4:**
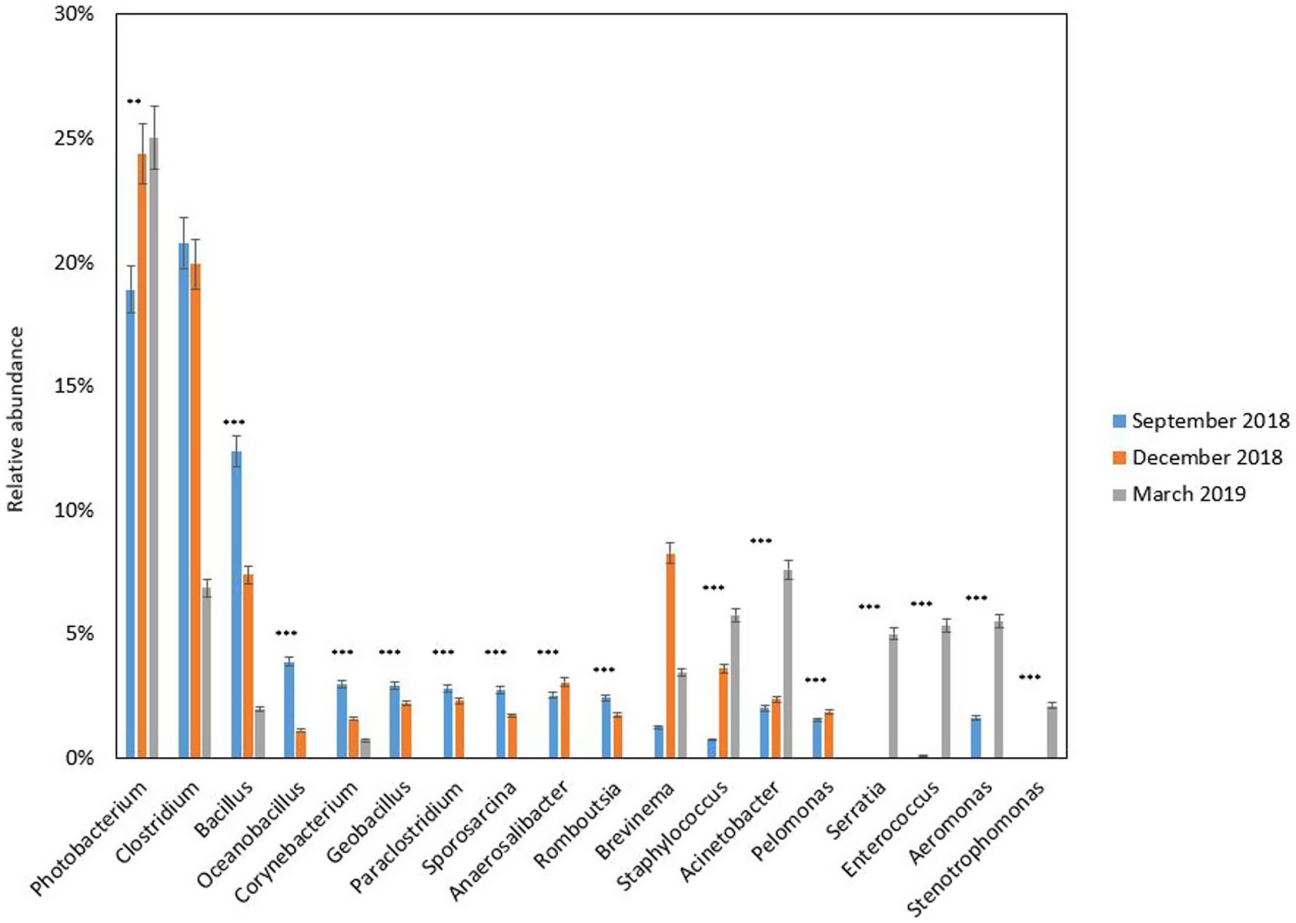
Relative abundance (%) of bacterial genera in the gut of freshwater Chinook salmon of the different sampling time points (September 2018, December 2018, and March 2019). Only the top 10 abundant genera in at least one sample from each sampling time point are shown. * = *p* value less than 0.05, ** = *p* value less than 0.01, and *** = *p* value less than 0.001.

Digesta samples were collected from the same cohort of fish at multiple time points, which allowed us to determine how the gut bacterial microbiome changed over time. The fish with fluid in their swim bladders were not included in the gut microbiota analysis. At the phylum level (Figure 2A), *Firmicutes* and *Proteobacteria* contributed the most reads across all sampling time points (88.43%). *Firmicutes* was predominant in September 2018 (61.66%) and December 2018 (53.80%), while *Proteobacteria* was more dominant in March 2019 (60.25%). Other abundant phyla (≥ 1%) were *Actinobacteria*, *Bacteroidetes*, *Fusobacteria,* and *Spirochaetes*. At the order level (Figure 2B), the gut microbiota was dominated by *Bacillales* and *Clostridiales* at early stages (56.08% in September 2018 and 47.48% in December 2018), while the relative abundance of *Vibrionales* (31.83%), *Lactobacillales* (12.32%) and *Enterobacteriales* (9.66%) were significantly increased by the end of sampling (March 2019). Taken together, *Proteobacteria* was the most diverse phylum mainly dominated by *Vibrionales* (40.25%) (Figure 3A). *Firmicutes* was dominated by *Bacillales* (39.65%), *Clostridiales* (34.54%), and *Lactobacillales* (22.75%), *Corynebacteriales* (45.85%), and *Flavobacteriales* (48.15%) were the most abundant orders in *Actinobacteria* and *Bacteroidetes,* respectively (Figure 3B). The differential abundance analysis of the top 10 abundant genera in each sampling time point revealed that *Clostridium* was the most abundant genus at the beginning of the trial (Figure 4). However, it became less dominant over time although the relative abundance change was not statistically significant *(p =* 0.192). The relative abundance of *Photobacterium* increased significantly from 18.89 to 24.38% (*p =* 0.008) and it became the most abundant genus by the end of sampling. The relative abundance of *Enterococcus*, *Serratia, Staphylococcus,* and *Stenotrophomonas*, were all lower than 1% at the beginning but had significantly increased to 5.33, 5.03, 5.77, and 2.13%, respectively, by the end of sampling. Other abundant genera with significant increases were *Aeromonas* and *Acinetobacter*. In contrast, *Bacillus*, *Oceanobacillus*, *Corynebacterium*, *Geobacillus*, *Paraclostridium, Sporosarcina, Romboutsia*, *Anaerosalibacter*, and *Pelomonas* significantly declined by the end of the sampling.

**Figure 5:**
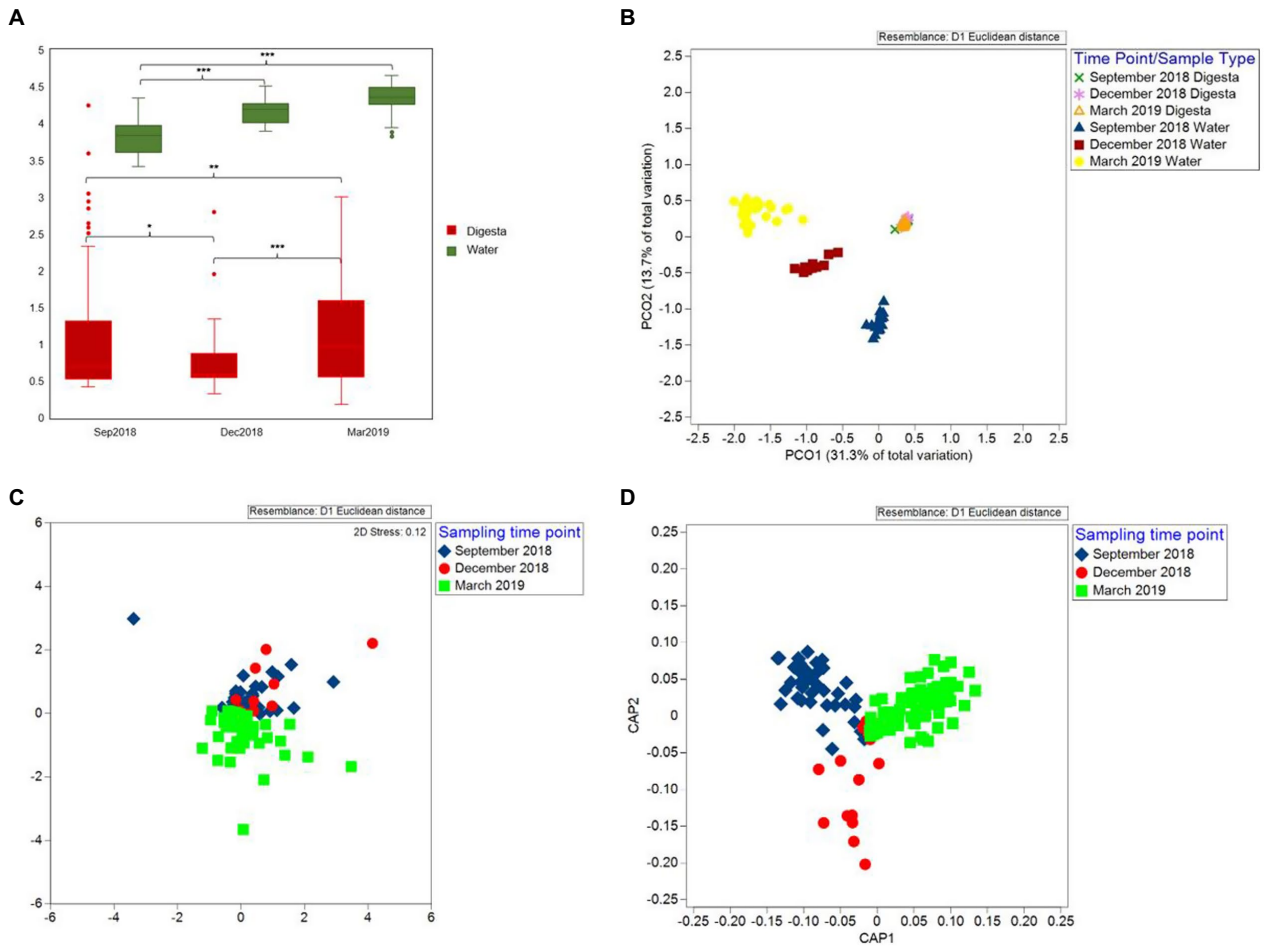
Alpha and beta diversity of gut and water microbiota during three sampling time points (September 2018, December 2018, and March 2019). **(A)** Shannon diversity of Chinook salmon gut microbiota and rearing water microbiota. The Shannon diversity of water microbiota was significantly higher than that of the gut microbiota regardless of the time points (*p* < 0.001). Additionally, between time-points groups, the Shannon diversity of water microbiota significantly increased from September 2018 to December 2018 (*p* < 0.001) but was stable from December 2018 to March 2019 (*p* = 0.799). For the gut microbiota, a decrease in the Shannon diversity from September 2018 to December 2018 (*p* = 0.054). However, the diversity significantly increased in March 2019 (*p* = 0.002). **(B)** Principal coordinates analysis (PCoA) plot shows that the water samples were highly distinguishable from digesta samples, and three different water clusters represented water sampled from different time points. **(C)** Non-metric multidimensional scaling (nMDS) plot. **(D)** Canonical analysis of principal coordinates (CAP) plot illustrate that the Chinook salmon gut microbiota varied between sampling time-point groups. * = *p* value less than 0.05, ** = *p* value less than 0.01, and *** = *p* value less than 0.001.

Shannon diversity analysis showed a decrease in gut microbial diversity from September 2018 to December 2018 (*p* = 0.054). However, the diversity significantly increased in March 2019 (*p =* 0.002) (Figure 5A). For beta diversity, the nMDS and CAP scatter plots illustrated that the Chinook salmon gut microbiota varied between the time points (Figures 5C,D) and the PERMANOVA test indicated these changes were significant (Pseudo-*F* = 6.538, *p =* 0.001).

### 3.4. Microbial differences between water and gut microbiota

To understand how microbial communities differ between the host and environment, we first assessed the diversity and composition of water microbiota. In contrast to the gut microbiota, the Shannon diversity of water microbiota was significantly higher regardless of the time points (*p <* 0.001) (Figure 5A). The Shannon diversity significantly increased from September 2018 to December 2018 (*p <* 0.001) but was stable from December 2018 to March 2019 (*p =* 0.799) (Figure 5A). For microbial similarity, PCoA plots showed that the water samples were highly distinguishable from digesta samples, and three different clusters represented water sampled from different time points (Figure 5B). PERMANOVA test further confirmed the variation statistically (Pseudo-*F* = 77.447, *p =* 0.001). For water microbial composition, the community was mainly composed of *Proteobacteria* (an average of 53.69%) and *Bacteroidetes* (an average of 29.29%) (Figure 2C). In contrast to the gut microbiota, the relative abundance of *Firmicutes* (an average of 0.45%) was much lower (< 1%). Due to the presence of many unclassified bacteria (relative abundance higher than 50% at the family level), we only further analyzed the composition to the order level (Figure 2D). The relative abundance of *Vibrionales* was comparatively lower in the water (an average of 1.73% among three sampling time points) compared to that in the gut (an average of 20.26% among three sampling time points).

**Figure 6:**
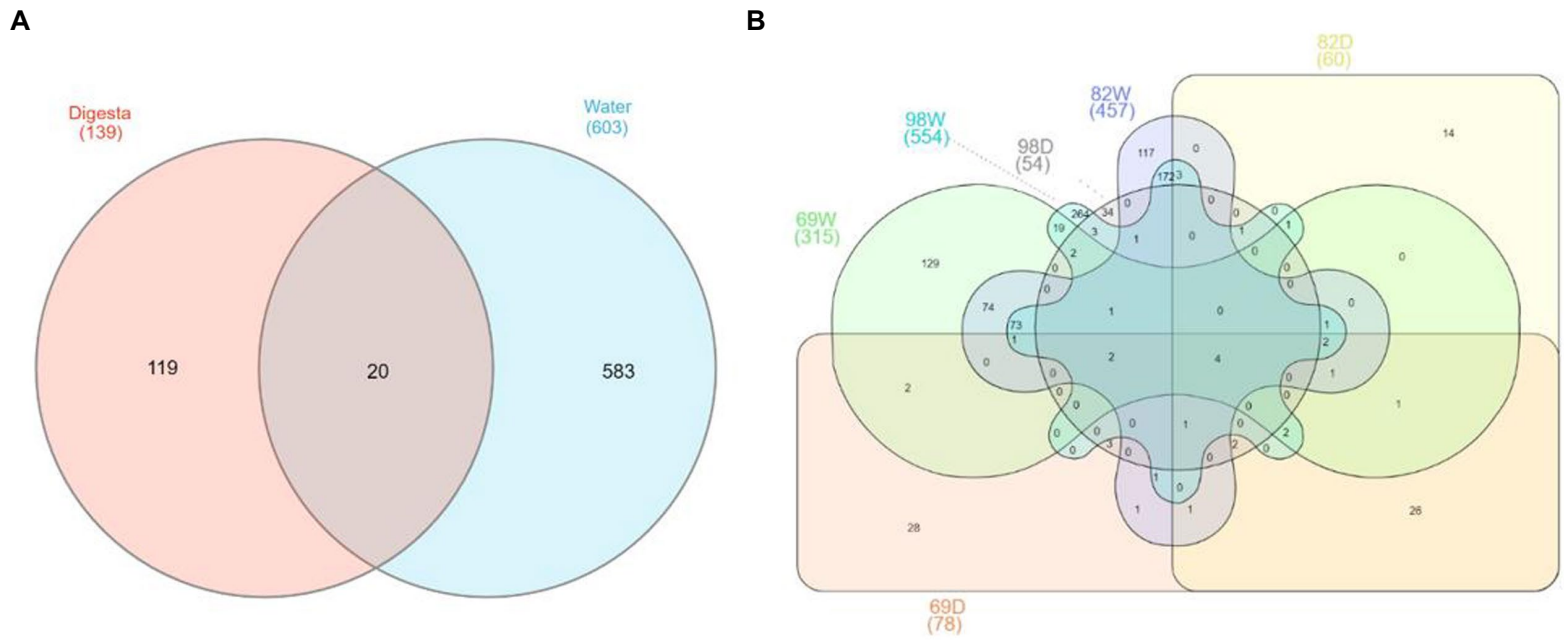
Venn diagram shows the shared and unique OTUs between the gut and water microbiota. **(A)** The common and unique OTUs between the gut and water microbiota. Overall, a total of 722 abundant OTUs (> 0.01% total reads) were collected between water and digesta, with 583 and 119 being unique to water and digesta, respectively, in addition to 20 being detected within both groups. **(B)** The shared and unique OTUs between the gut and water microbiota based on sampling time points. In September 2018, a total of 378 abundant OTUs were collected between water (69 W) and digesta (69D), with 300 and 63 being unique to water and digesta, respectively, and 15 being detected within both groups. In December 2018, a total of 504 abundant OTUs were collected between water (82 W) and digesta (82D), with 444 and 47 being unique to water and digesta, respectively, and 13 being detected within both groups. In March 2019, a total of 593 abundant OTUs were collected between water (98 W) and digesta (98D), with 539 and 39 being unique to water and digesta, respectively, and 15 being detected within both groups.

To understand the variance of OTUs between water and digesta, we assessed the top abundant OTUs (with reads >0.01%). Venn diagram illustrated that 20 OTUs were shared between digesta and water samples (Figure 6A). One hundred nineteen OTUs were only detected in the digesta and 583 OTUs were only detected in the water. We further compared the OTUs from water and digesta samples across the three sampling time points. Ultimately, only 4 OTUs (*Photobacterium piscicola*, *Brevinema* sp., *Photobacterium* sp., and *Acinetobacter* sp.) were shared overall (Figure 6B).

### 3.5. Core gut microbiota identification

To understand the core gut microbiota of freshwater farmed Chinook salmon, we selected the gut-derived OTUs of which prevalence was higher than 75% and relative abundance was higher than 1% independently at each sampling timepoint. Two OTUs were identified from the September 2018 cohort, followed by three OTUs from December 2019 cohort and one OTUs from March 2019 cohort (Supplementary Table 6). Taken together, we identified one OTU (OTU000018) that was assigned to the genus *Photobacterium* as the core gut microbiota for freshwater Chinook salmon. OTU000018 was the most abundant OTU, predominating at all time points, making up 56.68% of total reads. Notably, the predominance of OTU000018 increased during the experiment although the change was marginally significant (*p =* 0.021).

### 3.6. Correlation between microbiota and fish health

To evaluate how the gut microbiota may be related to salmon health and growth, we first investigated the relationship between intestinal mucous cell density and gut microbial diversity. No significant difference was found for either alpha (*p =* 0.510) or beta diversities (Pseudo-*F* = 1.347, *p =* 0.156) between the two mucous cell density groups. Furthermore, there was no significant correlation between Shannon diversity and mucous cell density using Pearson’s *R* analysis (*p =* 0.382). We also found no significant differences in alpha (*p =* 0.196) and beta diversities (Pseudo-*F* = 1.477, *p =* 0.066) between fish groups possessing different plasma biochemistry range values.

Additionally, we assessed how the gut microbiota was correlated with swim bladder fluid accumulation. In terms of alpha diversity, a reduced Shannon diversity was observed in the fecal microbiome of fish with swim bladders containing fluid (*p =* 0.006). The analysis of beta diversity showed that the gut microbial communities were significantly different in the fish with fluid accumulated their swim bladders (Pseudo-*F* = 5.191, *p <* 0.001).

For taxonomic analysis, the composition of abundant gut microbiota (> 0.01% of total reads) of fish with different swim bladder conditions was analyzed by differential abundance analysis at the genus level. Bacteria assigned to *Enterococcus* (*p <* 0.001), *Serratia* (*p =* 0.014) and *Aeromonas* (*p =* 0.0183) were more abundant in the fish with fluid in their swim bladders. The genera *Raoultella* and *Stenotrophomonas* were not detected during the sampling in December 2018. Fish with the two different swim bladder conditions shared 30 OTUs and 16 genera (Supplementary Table 7) and 15 OTUs and 7 genera were only detected in the fish with normal swim bladders. Fish with fluid in their swim bladders had 17 OTUs and 6 genera that were not found in fish with normal swim bladders.

### 3.7. Correlation between microbiota and fecal score and fish growth performance

The Shannon diversity was reduced from FS1 to FS3 (*p =* 0.025) but increased from FS3 to FS4 (*p =* 0.005). However, no significant difference was observed among these FS groups in terms of beta diversity (Pseudo-*F* = 0.931, *p =* 0.448). The gut microbiota of FS4 and the microbiota of surrounding water substantially overlapped in both terms of alpha (*p =* 0.083) and beta diversity (Pseudo-*F* = 5.744, *p =* 0.094). Additionally, no differences were evident in alpha (*p* = 0.454) and beta diversity (Pseudo-*F* = 0.924, *p* = 0.495) between the two growth performance groups.

### 3.8. Profile monitoring for microbial relationship alteration in digesta regarding different factors

**Figure 7:**
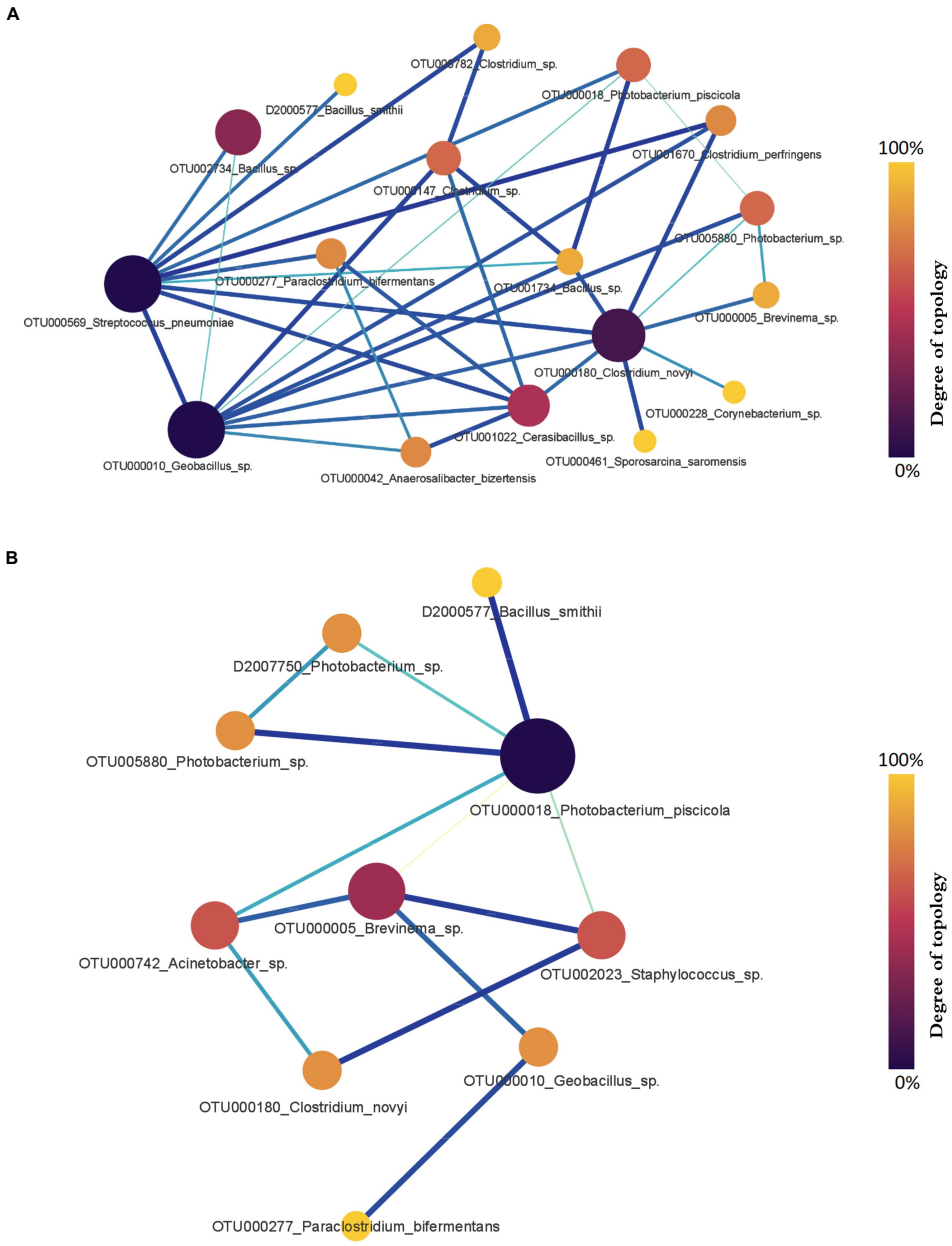
The gut microbial relationship alteration (RA) network for Chinook salmon based on sampling time points. **(A)** The RA network for Chinook salmon feces collected in September 2018 and December 2018. *Clostridium*, *Streptococcus*, and *Geobacillus* were the top three genera with the largest degrees of topology. **(B)** The RA network for Chinook salmon feces collected in December 2018 and March 2019. *Photobacterium*, *Brevinema*, *Staphylococcus* were the top three genera with the largest degrees of topology. The color and size of nodes represent the degree of topology in the network, and the edge width is proportional to the value of PM scores.

Comparing microbial profiles under different growth and health statuses using the PM2RA method provided additional insights into the salmon gut microbial interactions including those associated with possible dysbiosis (e.g., fish with abnormal swim bladders). First, we evaluated the RA in the microbiota between salmon sampled at different time points. Between the fish collected in September 2018 and December 2018, 11 hub genera with significant abundance changes (*p* < 0.05) were found to be involved in the RA network (Figure 7A). The top three genera with the largest degrees of topology were *Clostridium*, *Streptococcus,* and *Geobacillus*. Between the fish collected in December 2018 and March 2019, a RA network containing nine hub genera with significant abundance changes (*p* < 0.05) was observed (Figure 7B). *Photobacterium*, *Brevinema*, *Staphylococcus,* and *Acinetobacter* had high interconnectedness. However, the RAs between bacteria assigned to *Clostridium* and *Photobacterium* were not significantly changed, and taxa related to these two genera were different. The highest PM score related to *Clostridium* was observed between OTU006782 assigned to *Clostridium* and OTU000596 assigned to *Streptococcus* (PM = 0.964) (Figure 7A). The highest PM score related to *Photobacterium* was observed between OTU000018 assigned to *Photobacterium* and D2000577 assigned to *Bacillus* (PM = 0.965) (Figure 7B).

**Figure 8:**
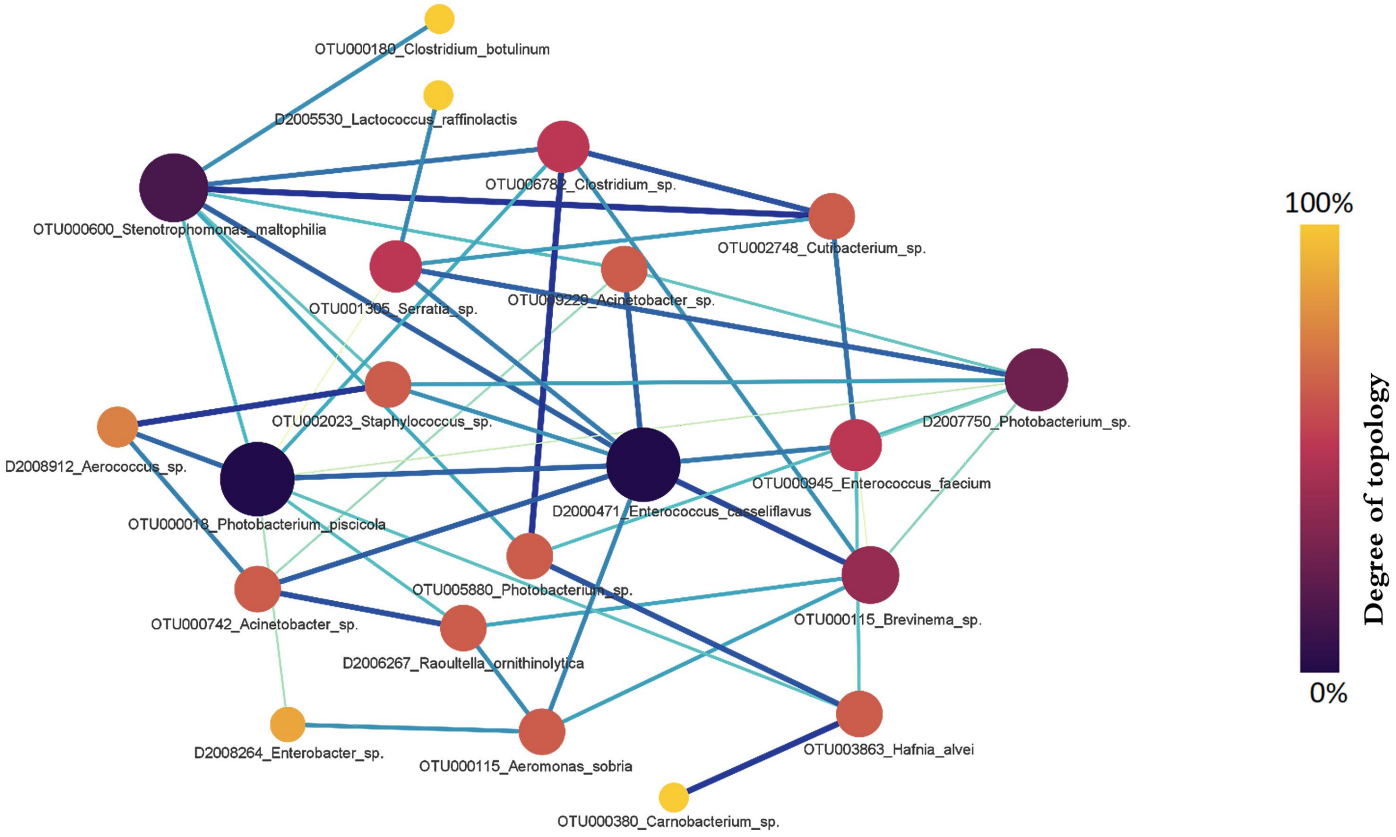
The gut microbial relationship alteration (RA) network for Chinook salmon based on swim bladder status (fluid present = 36, no fluid = 42). *Photobacterium*, *Enterococcus*, and *Stenotrophomonas* were the top three genera with the largest degrees of topology. The colour and size of nodes represent the degree of topology in the network, and the edge width is proportional to the value of PM scores.

Next, we assessed the RA in the microbiota between salmon with and without fluid in their swim bladders. Sixteen hub genera were involved in the RA network (Figure 8). *Photobacterium*, *Enterococcus,* and *Stenotrophomonas* were the top three genera with the largest degrees of topology (Figure 8). Specifically, OTUs assigned to *Enterococcus* and *Photobacterium* were the active OTUs with the largest degrees of topology, followed by OTUs assigned to *Stenotrophomonas* and *Brevinema*. The RA between OTUs assigned to *Photobacterium* (OTU000018) and *Enterococcus* (D2000471) (PM = 0.886) and OTUs assigned to *Photobacterium* (OTU000018) and *Stenotrophomonas* (OTU000600) (PM = 0.73) changed significantly.

## 4. Discussion

### 4.1. Overall gut microbiota of freshwater farmed Chinook salmon

For freshwater farmed Chinook salmon, their gut microbial composition at the phylum level was consistent with other freshwater teleosts, such as carp (Eichmiller et al., 2016; Luo et al., 2022), yellow catfish (*Pelteobagrus fulvidraco*) (Li et al., 2014), and zebrafish (*Danio rerio*) (Roeselers et al., 2011). In general, *Firmicutes* and *Proteobacteria* were most abundant, followed by *Actinobacteria, Bacteroidetes, Fusobacteria,* and *Spirochaetes*. The dominance of *Firmicutes* and *Proteobacteria* was also observed in previous studies of other salmonids sampled over a range of conditions (Ingerslev et al., 2014; Zarkasi et al., 2014; Gajardo et al., 2016; Llewellyn et al., 2016). As an extension of our previous study (Zhao et al., 2021), we tracked Chinook salmon gut microbiota changes in a freshwater RAS at 17°C over time and found that the most abundant phyla changed with fish growth from *Firmicutes* to *Proteobacteria,* although the taxonomic distribution of the overall microbiota was stable. This result suggests that the composition of gut microbiota and predominant microbial taxa changed with Chinook salmon development as indicated previously for both farmed and wild salmon (Llewellyn et al., 2016; Zhao et al., 2020). The volume of the fish intestine increases during the host’s development (from early to adult stages), which may contribute to a more extensive and stable habitat for diverse microbial communities (Yan et al., 2012). Additionally, a greater volume of digesta due to an increased intestinal capacity could contribute to a more stable community structure but this also depends on gut residence time which can vary significantly. This effect on gut communities has been noted in previous publications (Zhang et al., 2018; Piazzon et al., 2019). For Chinook salmon, this has also been previously observed where older fish gut community structure diverges from that associated with younger fish (Zhao et al., 2020).

Compared to previous studies on the gut microbiota of salmon sampled from natural environments (Hovda et al., 2012; Llewellyn et al., 2016), our study suggests that fish age plays a major role in affecting the gut microbial community when rearing the fish in a relatively stable environment (freshwater RAS). The variation in the gut microbial composition and diversity at different sampling time points suggests a clear correlation to the normal microbial succession, which has been reported in human (Martino et al., 2022) and other aquatic animals (Xiong et al., 2020; Xiao et al., 2021). It has been demonstrated that human gut microbiota changes can be deliberately modified across time, which differs from the human genome that is encoded at birth and cannot be altered during life (at least with current technology) (Martino et al., 2022). A recent study on gut microbiota interactions in zebrafish indicates that the increasing gut microbial stability is determined by the development of the immune system and the greater stability of nutrient absorption (Xiao et al., 2022). Furthermore, the change of feeding habit, including the use of probiotics, is also shown to affect the fish gut microbiota, especially at the early life stages (Deng et al., 2021; Vargas-Albores et al., 2021). However, the mechanism of probiotics colonization is still unclear as it is affected by fish physiology and genetic backgrounds (Merrifield et al., 2010; Ciric et al., 2019). A study on farmed post-smolt Chinook salmon shows that although the salmon is fed with a well-known probiotic strain (*Pediococcus acidilactici* strain MA 18/5 M), the strain is not able to colonize the gut and quickly declines when salmon are not actively consuming the probiotic (Ciric et al., 2019). By understudying the process of gut microbiota change during life stages, we may better understand how to manage the gut microbiota over time and in relation to fish health. The variation in microbiota with sampling-time points may be attributed to the host maturation that could involve more extensive microbial interactions within the intestine and between the host and the environment. Extensive histological and other functional analyzes are needed to evaluate the relationship between microbial transformation and GI tract development.

### 4.2. Chinook salmon gut microbiota significantly differed from water microbiota

We found that gut microbiota composition was significantly different from that of water microbiota with only 4 OTUs (two assigned to *Photobacterium*, one assigned to *Brevinema* and one assigned to *Acinetobacter*) shared overall during three sampling points. Based on earlier studies (Sullam et al., 2012; Wong and Rawls, 2012; Liu Q. et al., 2021; Zhao et al., 2021), we hypothesized that Chinook salmon harbors a relatively stable gut microbiota that is distinguishable from environmental microbiota. Although *Proteobacteria* dominated both communities, the microbial composition was different at the order level. Consistent with previous studies (Zhao et al., 2020, 2021), we detected high abundance of *Clostridiales*, *Bacilli*, *Lactobacilli*, and *Corynebacteriales* bacteria in the gut. A typical characteristic of all these bacterial orders is their preference for an anaerobic environment like that found in fish intestines, suggesting that most bacteria detected in fish guts represent symbionts and commensals instead of a passive collection of water bacteria (Sullam et al., 2012). It should be noted that some of these genera, such as *Geobacillus*, are feed-associated based on their thermophilic growth temperature requirements that range from 35 to 75°C (Zarkasi et al., 2016; Karlsen et al., 2022). Additionally, the gut microbial communities detected in our study mainly represent the allochthonous microbiota, which are passing through the lumen with food and digesta (Romero et al., 2014). Several studies have investigated the autochthonous microbial communities that have successfully colonized the fish intestine and demonstrate these populations are involved in host-environment microbial interactions (Kononova et al., 2019; Nyholm et al., 2022).

### 4.3. The core gut microbiota for freshwater Chinook salmon

The core gut microbiota for fish has been determined in many studies (Kokou et al., 2019), including Chinook salmon (Zhao et al., 2020, 2021; Steiner et al., 2021, 2022). However, the core microbial communities can vary due to different fish species, sampling points, and screening criteria. Regardless of health and growth variation, we detected a distinct dominance of *Photobacterium* spp. in the Chinook salmon gut. *Photobacterium* was also the only genus that occurred at the three sampling time points in our study. This is consistent with other studies demonstrating *Photobacterium* as one of the common members of fish intestinal microflora (Gajardo et al., 2016; Llewellyn et al., 2016). Persistent high FS values have been proposed to be indicative of fish with a GI microbiome imbalance, possible dysbiosis and/or association with poor feeding rates (related as reduced weight gain) (Zarkasi et al., 2016). We hypothesized that the high microbial diversity in FS4 may be related to a high proportion of water in the gut since the water microbial community had a relatively higher alpha diversity. We found that bacteria assigned to *Clostridium* were more predominant in September 2018, while bacteria assigned to *Photobacterium* were more predominant in December 2018 and March 2019. Taken together, our results indicate that *Photobacterium* sp. may be a native intestinal bacterium for farmed Chinook salmon when the salmon are reared in freshwater with higher abundance occurring in older fish. More studies are needed to investigate the characteristics of *Photobacterium* since it could closely interact with the host and other abundant gut community members and thus may play a key role in host performance (Steiner et al., 2022).

### 4.4. Interactions between gut microbiota, salmon health, and growth performance

Although we included only apparently healthy fish based on their external appearance, almost half of the salmon sampled in March 2019 (36 out of 78) had fluid in their swim bladders. Besides inflammatory reactions, fluid accumulation is one of the main swim bladder disorders in fish and it has been encountered in ornamental fish, such as koi carp (*Cyprinus carpio*) (Sirri et al., 2020). Physostomous fish like Chinook salmon have their swim bladders connected to the foregut, more specifically with the esophagus and stomach *via* a short pneumatic duct (Sado et al., 2020). Physostomous fish need to refill their swim bladder periodically by swallowing air at the surface, which permits the gas to enter into or be released through the alimentary canal (Stewart and Hughes, 2014). Due to the specific anatomic structure of the pneumatic duct, microorganisms in the digestive tract can potentially enter the duct and migrate into the swim bladder (Sirri et al., 2020). By detecting a reduced microbial diversity, a different microbial composition, and the presence of opportunistic pathogens, we suspected that a potential dysbiosis occurred in Chinook salmon with fluid in their swim bladders. A recent study showed that farmed rainbow trout in a freshwater RAS shared their gut microbiota with swim bladder-associated microbiota (Villasante et al., 2019). The core swim bladder-associated bacteria identified for rainbow trout, including *Photobacterium*, *Clostridium*, *Bacillus,* and *Streptococcus* (Villasante et al., 2019), were also detected predominantly in the GI tract in this study. Swim bladder microbiota was not analyzed in our study, so the connection between dysbiosis and fluid in the swim bladder is only hypothesized. Future studies are recommended to further investigate the mechanism of swim bladder fluid accumulation and the interaction between microbial communities in the gut and the swim bladder in Chinook salmon.

The gut microbiota of Chinook salmon with fluid in their swim bladder showed a high relative abundance of *Enterococcus*. For the aquaculture industry, some species of *Enterococcus* is often regarded as a potential probiotic that can be added to aquafeed because of its high tolerance to acidic pH, adherence to the GI tract, immune-modulatory activity, and antagonistic activity to entero-pathogens (Akbari et al., 2021). However, Timur (2019) demonstrated that *E. casseliflavus* was correlated with diseased juvenile meagre (*Argyrosomus regius*) from inland-based facilities and the related syndromes were reobserved when a recovered isolate was injected intraperitoneally. Due to the high relative abundance of *Enterococcus* observed in our experimental fish, we hypothesize that the occurrence of *Enterococcus* was associated with potential gut dysbiosis and might potentially impact the functioning of the swim bladder in farmed Chinook salmon.

There was a high relative abundance of *Aeromonas* spp. in fish with fluid in their swim bladders. Many species within the genus *Aeromonas* have been implicated in mortality, resulting in losses estimated in millions of dollars and increasing cost of fish production to the aquaculture industry (Fečkaninová et al., 2017). *A. sobria* has been reported to cause Motile *Aeromonas* Septicemia (MAS) in zebrafish (Pullium et al., 1999) and striped catfish (*Pangasianodon hypophthalmus*) (Le et al., 2018); *A. rivipollensis* has been isolated from wild nutria (*Myocastor coypus*) and is regarded as a potential zoonotic pathogen (Park et al., 2018); *A. veronii* is known to have virulence factors capable of causing freshwater fish diarrhea and ulcer syndrome (González-Serrano et al., 2002; Li T. et al., 2020). Particularly, *A. hydrophila* has been associated with swim bladder infections in wild freshwater fish (Teskeredžić et al., 2000) and has been associated with gastric dilation and air sacculitis (GDAS) in Chinook salmon farmed in the marine environment (Lumsden et al., 2002). Referring to previous studies (Lumsden et al., 2002; Forgan and Forster, 2007a,b), it is possible that the diet retention properties may have been an issue in this trial and could have led to swim bladder fluid accumulation, even though the trial was in freshwater.

High relative abundance of genera *Stenotrophomonas* and *Raoultella* was detected in the fish with abnormal swim bladders. Previous studies revealed the correlation of *S. maltophilia* with multidrug resistance in diseased yellowtail (*Seriola quinqueradiata*) (Furushita et al., 2005) and bacterial infections in freshwater ornamental fish (Musa et al., 2008). *R. ornithinolytica* can convert histidine to histamine, which leads to scombroid poisoning in humans (Hwang et al., 2020). However, there is limited information on the pathology of *Raoultella* in commercial fish. The decreasing abundance of *Photobacterium* and the increasing abundance of *Enterococcus* and *Stenotrophomonas* may indicate an antagonistic relationship between these taxa.

Microorganisms are essential for the development and differentiation of mucous cells (Bates et al., 2006). Our study indicates that microbial diversity is not correlated to GI tract mucous cell density. This result is in contrast to a recent finding that elevated GI mucous cell numbers were positively correlated with gut microbial richness in juvenile Atlantic salmon collected from freshwater hatcheries (Minich et al., 2020). Since abiotic factors, such as diet, temperature, and salinity, were fully controlled in this study, we suspect that biotic factors, such as fish species and development stage, may affect intestinal mucous cell density. Fish infected by specific pathogens can have different gut microbial communities, which should be considered when interpreting results (Wang C. et al., 2018). Several studies have evaluated host–microbe interactions by using germ-free and gnotobiotic models (Darnaud et al., 2021; Pérez-Pascual et al., 2021; Luna et al., 2022; Renu et al., 2022), but detailed mechanisms of how the fish microbiota interact with mucosal surfaces need to be further investigated.

Although 16S rRNA gene sequencing has enlarged the understanding of microorganisms in humans, mammals and fish, its low taxonomic resolution is a limitation. It is not as informative or powerful for identifying the bacteria at species- or strain-specific levels. All OTUs were identified based on the Silva non-redundant 16S rRNA database and more advanced sequencing technologies should undergo further verification of their taxonomical definition, such as the species level. For example, we detected a high relative abundance of bacteria assigned to *Serratia* and *Brevinema*, but it was not possible to link specific bacterial species to Chinook salmon performance. Likewise, over 50% of the reads in water samples could not be identified to the genus level. Therefore, although the analysis of relative abundance and microbial relationship alterations *via* 16S rRNA sequencing can provide insights for pathogen targeting, more studies are needed to better understand the pathogenicity of these versatile microorganisms and elaborate efficient and accurate measures to distinguish harmful and beneficial probiotic strains.

## 5. Conclusion

In conclusion, our results indicated that *Photobacterium* spp. was the core gut microbiota for freshwater farmed Chinook salmon and its interaction with other microbes was important for fish health management. The gross analyzes of microbial relationship alterations and relative abundance changes indicated that genera *Enterococcus*, *Aeromonas* and *Stenotrophomonas* were associated with the presence of fluid in the swim bladder of Chinook salmon. Our findings increase the knowledge of the temporal dynamics of the gut microbe in farmed fish and help the industry utilize the gut microbiota as a potential health indicator for modern aquaculture. Advanced sequencing techniques, histological and microbiological measurements, and a more in-depth understanding of resident gut microbiota and their properties are strongly recommended to differentiate beneficial and harmful bacterial strains.

## Data availability statement

The data is deposited in the NCBI Sequence Read Archive under accession code SRP348807 (PRJNA 781774).

## Ethics statement

The animal study was reviewed and approved by University of Tasmania Animal Ethics Committee.

## Author contributions

JS, SW, KS, BN, JB, CC, and RZ: conceptualization and methodology. RZ and JB: data curation and formal analysis and investigation. JS, SW, KS, BN, JB, and CC: resources, writing–review, and editing. RZ: writing–original draft preparation. JS, SW, and KS: funding acquisition. JS, BN, JB, and CC: project administration. BN, JB, and CC: supervision. All authors contributed to the article and approved the submitted version.

## Funding

This study was funded by the New Zealand Ministry of Business, Innovation and Employment (MBIE) Efficient Salmon research program [CAWX1606] and is a collaboration between the University of Tasmania, Australia and the Cawthron Institute, New Zealand.

## Conflict of interest

The authors declare that the research was conducted in the absence of any commercial or financial relationships that could be construed as a potential conflict of interest.

## Publisher’s note

All claims expressed in this article are solely those of the authors and do not necessarily represent those of their affiliated organizations, or those of the publisher, the editors and the reviewers. Any product that may be evaluated in this article, or claim that may be made by its manufacturer, is not guaranteed or endorsed by the publisher.

## Footnotes

^1^ https://www.ianz.govt.nz/

^2^ https://www.ifcc.org/

^3^ http://www.pm2ra-xingyinliulab.cn/

^4^ https://cytoscape.org/
